# Aldosterone-progesterone relationship in sexually intact Chihuahua bitches

**DOI:** 10.1186/s12917-023-03704-2

**Published:** 2023-09-05

**Authors:** Alberto Galizzi, Greta Dossi, Vitaliano Borromeo, Paola Pocar, Debora Groppetti, Chiara Locatelli

**Affiliations:** Department of Veterinary Medicine and Animal Science, University of Milan, Via dell’Università 6, Lodi, 26900 Italy

**Keywords:** Dog, Aldosterone, Progesterone, Sex, RAAS, MMVD

## Abstract

**Background:**

Aldosterone represents an important target of heart failure therapy and may be a valuable indicator of the renin-angiotensin-aldosterone system activity. However, its assessment might be challenging because of the effect of individual factors. In a recent study, intact female dogs showed the highest value of urinary aldosterone-to-creatinine ratio (UAldo:C) compared to other sex categories. In humans and rodents, an influence of progesterone has been reported by several studies. To our knowledge, the relationship between aldosterone and progesterone has not yet been investigated in dogs. The aim of this prospective study was to investigate this relationship in sexually intact Chihuahua females, measuring both hormones twice in the same bitch, that is in anoestrus when progesterone concentrations are baseline and in dioestrus when they are high.

**Results:**

The study population consisted of 14 sexually intact Chihuahua bitches. Serum progesterone (34.06 (21.17–44.90) vs. 0.19 [0.13–0.38] ng/ml; P < 0.001) and urinary aldosterone (9886.98 ± 5735.22 vs. 5005.72 ± 2127.73 pg/ml; P = 0.01) were significantly higher in dioestrus compared to anoestrous. Urinary aldosterone-to-creatinine ratio was higher in dioestrus compared to anoestrus (4.16 [3.17–6.80] vs. 3.39 ± 1.64 µg/g), but it did not reach the statistical significance (P = 0.056). Serum progesterone showed a moderate positive correlation with urinary aldosterone (ρ = 0.638, P < 0.001) and UAldo:C (ρ = 0.516, P = 0.005).

**Conclusions:**

The results of the present study suggest the existence of a progesterone-aldosterone relationship in canine species, indicating that sex and phase of reproductive cycle should be taken into account when interpreting aldosterone concentrations. Further studies are needed to confirm these results on a larger canine population and to identify the underlying mechanisms in this species.

## Introduction

Chronic renin-angiotensin-aldosterone system (RAAS) activation promotes and perpetuates the congestive heart failure (CHF) syndrome [1, 2]. Prolonged and excessive aldosterone secretion leads to several harmful cardiovascular effects (e.g., volume and pressure overload, inflammation, fibrosis), and aldosterone has become an important target of CHF therapy [1, 3, 4, 5, 6]. The assessment of RAAS activity in dogs with heart diseases could improve their clinical and therapeutic management. Since aldosterone represents the terminal hormone of the cascade, its assessment covers the RAAS alternative pathways and the aldosterone breakthrough phenomenon [1, 7, 8]. Thus, it holds potential as a valuable indicator of a patient’s overall RAAS activity. However, the interpretation of this marker might be challenging. In a recent study in dogs, aldosterone levels appeared to be affected by individual factors. In particular, Chihuahuas and sexually intact females showed the highest values of urinary aldosterone-to-creatinine ratio (UAldo:C) among breed and sex categories respectively [9]. The interbreed variability might be related to the polymorphisms of genes encoding for RAAS components, as previously suggested [8, 10]. Regarding gender, RAAS activity has been suggested to be affected by sexual hormones. A rise in aldosterone levels have been reported to occur during the luteal phase of menstrual cycle in women and progesterone has been found to be a high affinity antagonist for human mineralocorticoid receptors (MR), preventing aldosterone interaction. Thus, the increase in serum and urinary aldosterone levels likely occurs because of MRs occupation and compensatory activation of RAAS [11, 12, 13]. However, progesterone might contribute to the rise of aldosterone concentration even through RAAS-independent mechanisms, such as directly stimulating aldosterone production from zona glomerulosa [11, 14]. In dogs, an increase in RAAS activity during pregnancy has been suggested [15, 16], but, to our knowledge, the relationship between progesterone and aldosterone levels has not yet been investigated in this species. The aim of this prospective study was to investigate the relationship between progesterone and aldosterone in sexually intact Chihuahua bitches, measuring these hormones in anoestrus and dioestrus (i.e., low and high progesterone state respectively).

## Results

### Animals

The study population consisted of 14 sexually intact Chihuahua females. The mean age was 3.71 ± 1.55 years and the mean body weight was 2.82 ± 0.41 kg; 6 out of 14 dogs had a Body Condition Score (BCS) of 4/9 and 8 out of 14 dogs had a BCS of 5/9. All dogs were fed a normal-sodium diet (0.34% [OASY One Animal Protein Adult/Small Mini Salmon; OASY, Republic of San Marino] and 0.4% sodium [Royal Canin Mini Adult; Royal Canin, Aimargues, France]). The mean systolic arterial pressure was 135.14 ± 16.92 mmHg. Complete blood count (CBC), biochemical profile and urinalysis were unremarkable in all dogs. Cardiac auscultation was unremarkable in 11/14 dogs; 2 dogs presented a I/VI grade left apical systolic murmur and 1 dog an II/VI grade left apical systolic murmur. Echocardiography was unremarkable in 11 dogs (American College of Veterinary Internal Medicine [ACVIM] stage A), while 3 dogs were diagnosed with Myxomatous Mitral Valve Disease (MMVD) ACVIM stage B1^17^. Dogs in dioestrus were evaluated between 23 and 48 days after the onset of serosanguinous vulvar discharge. Dogs in anoestrus were evaluated between 99 and 302 days after the last oestrus and before the subsequent proestrus. Phases of reproductive cycle (anoestrus and dioestrus) were confirmed by vaginal cytology in all dogs.

### Hormone measurements

Results of hormone measurements are reported in Table 1. Serum progesterone and urinary aldosterone were significantly higher in dioestrus compared to anoestrous. Urinary aldosterone-to-creatinine ratio was higher in dioestrus compared to anoestrus, but it did not reach the statistical significance. N-Terminal pro-B-type natriuretic peptide (NT-proBNP) was not significantly different between anoestrous and dioestrus.

**Table 1:** Hormones measurements in anoestrus and dioestrus

	Anoestrus	Dioestrus	P value
Serum progesterone (ng/ml)	0.19 (0.13–0.38)	33.39 ± 14.87	< 0.001*
Urinary aldosterone (pg/ml)	5005.72 ± 2127.73	9886.98 ± 5735.22	0.010*
UAldo:C (µg/dl)	3.39 ± 1.64	4.16 (3.17–6.80)	0.056
NT-proBNP (pmol/l)	332.86 ± 163.50	394.50 (125-643.75)	0.139

UAldo:C urinary aldosterone-to-creatinine ratio, NT-proBNP N-Terminal pro-B-type natriuretic peptide

Normally distributed variables are presented as mean ± standard deviation. Non-normally distributed variables are presented as median and interquartile range (IQR)

*Significant difference (P value < 0.05) between anoestrus and dioestrus

In total sample (anoestrus + dioestrus), serum progesterone showed a positive moderate correlation with urinary aldosterone (ρ = 0.638, P < 0.001) and UAldo:C (ρ = 0.516, P = 0.005). N-Terminal pro-B-type natriuretic peptide was not significantly different between anoestrus and dioestrus and no significant correlations were found between NT-proBNP and serum progesterone, urinary aldosterone and UAldo:C.

### Urinalysis

Results of urinalysis are reported in Table 2. Urinary protein-to-creatinine ratio (UP/UC) was significantly higher in anoestrus compared to dioestrus. Urinary creatinine (UC) was significantly higher in dioestrus compared to anoestrus. No significant differences in urine specific gravity (USG) and urinary protein (UP) were found between the two phases of oestrus cycle.

**Table 2:** Comparison of urinary parameters between anoestrus and dioestrus

	Anoestrus	Dioestrus	P value
Urinary creatinine (mg/dl)	150.94 ± 26.95	194.54 ± 57.39	0.008*
Urinary protein (mg/dl)	35.64 ± 9.29	33.29 ± 12.05	0.488
UP/UC	0.24 ± 0.063	0.17 ± 0.042	0.003*
USG	1060 (1048.75–1062)	1059.07 ± 10.93	0.327

UP/UC urinary protein-to-creatinine ratio, USG urine specific gravityNormally distributed variables are presented as mean ± standard deviation. Non-normally distributed variables are presented as median and interquartile range (IQR)

*Significant difference (P value < 0.05) between anoestrus and dioestrus

## Discussion

The results of the present study suggest the existence of a progesterone-aldosterone relationship in canine species.

As expected, progesterone significantly increased in dioestrus compared to anestrus [17]. Urinary aldosterone showed a significant parallel increase and UAldo:C raised in dioestrus with a P value close to significance (0.056). Moreover, urinary aldosterone and UAldo:C showed a significant positive correlation with progesterone in total population.

In women, the luteal phase of the menstrual cycle is characterized by increased levels of progesterone and oestradiol, and a rise in aldosterone concentration during this period has been reported by several studies [11, 12, 18–20]. Progesterone represents a high-affinity antagonist of MRs in humans and rats, acting as a competitive inhibitor of aldosterone and inducing natriuresis [13, 14, 18, 21–24]. Because of these properties, high levels of progesterone likely lead to an increase in aldosterone concentrations because of the receptor occupation and the compensatory activation of the RAAS [11, 19, 20, 24, 25, 26]. However, even a RAAS-independent mechanism has been suggested. Szmuilowicz et al. (2006) reported an increase in aldosterone levels during the luteal phase in women without a concurrent increase in plasma renin activity and angiotensin-II [11]. In the same study, they found that the incubation of rat zona glomerulosa cells with progesterone caused a significant increase in aldosterone production compared to the incubation with vehicle alone [11]. A similar result has been obtained with human adrenocortical cells [27]. These findings suggest that progesterone directly stimulate adrenal aldosterone production. Progesterone receptors has been found in the adrenal capsular cells of female mice [28] and in human adrenocortical cells [27], and it has been hypothesized that this hormone might influence aldosterone secretion through its own receptors [11]. A second hypothesis is that increased levels of progesterone correspond to increased levels of substrate for 21-hydroxylase in the adrenal cells, since progesterone represent a precursor in the aldosterone biosynthetic pathway [11, 29]. A similar mechanism has been already hypothesized for the positive relationship between progesterone and cortisol reported in peripartum bitches and naturally cycling women [26, 30]. However, the role of progesterone as a direct stimulator of aldosterone secretion remains subject of debate, since conflicting results have been reported in vitro [31].

Interestingly, progesterone has different properties in other species. In cartilaginous and ray-finned fish, in which aldosterone is not synthetized, progesterone represents a physiological activator of MRs [13]. Mineralocorticoid receptors preceded aldosterone by millions of years, and they evolved from an ancestral protein in common with glucocorticoid, progesterone and androgen receptors. Aldosterone appeared for the first time in lungfish, which represent a transition from aquatic to terrestrial life [13, 32]. The role of progesterone as MR activator was lost in amphibians, alligators, rodents and humans [13]. Unexpectedly, progesterone activates MR in chicken and the reason remains unknown at this time, since the receptor has molecular characteristics similar to that of humans [13, 33]. Currently, a mutant human MR represents the only other case of MR activated by progesterone in terrestrial vertebrates [34]. In dogs, previous studies suggested an increase in RAAS activity in relation to pregnancy/oestrous cycle [15, 16, 19], but, to our knowledge, this is the first study that investigated the relationship between progesterone and aldosterone in canine species. Such as humans and rodents, dogs belong to mammals class and, from an evolution perspective, it could be presumed that similar mechanisms regulate the interactions among MR, progesterone and aldosterone. However, Johnson et al. (1970) found that natriuresis did not occur after progesterone infusion in healthy dogs [35], raising doubts about the role of progesterone as MR antagonist in this species. The underlying mechanisms are still to be determined and further investigations are warranted in dogs, especially in the molecular and cellular field.

Szmuilowicz et al. (2006) showed a direct correlation between aldosterone and progesterone among women receiving a high-sodium diet, but no differences in aldosterone levels between follicular and luteal phase were found among women in a low-sodium regimen [11]. Although these results confirm that progesterone is responsible of aldosterone increase, they also indicate that natremia remains a primary modulator of RAAS activity and it likely overwhelms the role of progesterone in case of sodium restriction [11]. In the present study, all dogs included were fed two type of normal-sodium commercial diet throughout the reproductive cycle and their sodium content was superimposable. Thus, it can be excluded that sodium intake influenced the difference in aldosterone levels between anoestrus and dioestrus in the present study.

In a previous study, Chihuahua showed significantly higher UAldo:C compared to other breeds [9]. Polymorphism of angiotensin converting enzyme has been reported in dogs, with wide prevalence variability among breeds 36. This gene variant has been associated with higher aldosterone levels and aldosterone breakthrough incidence in dogs [8]. In the present study, Chihuahua breed was chosen as inclusion criterion in order to eliminate the breed-related variability of aldosterone. It cannot be excluded that also the relationship between aldosterone and sexual hormones may be breed-related, and further investigation on multiple breeds are warranted.

Urinary aldosterone-to-creatinine ratio was higher in dioestrus compared to anoestrus, but it was not statistically different (P = 0.056) between the two phases. However, the following aspects should be taken into consideration: a small sample size (i.e., higher probability of type-II error) and the fact that urinary creatinine was significantly higher in dioestrus compared to anoestrus, in face of a superimposable USG. Urinary creatinine is one of the most-used correction factors to adjust several urinary biomarkers for dilution in spot urine samples [37]. The rationale is that UC excretion is constant [38]. However, creatinine clearance and UC excretion might be influenced by physiological changes [39]. For example, these parameters appeared significantly higher in mid-luteal phase (high oestrogen and high progesterone state) compared to pre-ovulatory phase (high oestrogen and low progesterone state) in women [40]. Variations of urinary creatinine, creatinine clearance and glomerular filtration rate have been reported also over the course of pregnancy [39, 41, 42]. Thus, it cannot be excluded that changes in UC excretion between anoestrus and dioestrus may have affected the UAldo:C results in the present study. To our knowledge, there are no reports about the variability of UC excretion over the reproductive cycle in dogs and further investigations are warranted to confirm this hypothesis.

N-Terminal pro-B-type natriuretic peptide was not significantly different between anoestrus and dioestrus and did not show any correlation with progesterone. Previous studies in dogs reported significantly higher NT-proBNP in intact females than intact males [43, 44], in agreement with studies in people, where women showed significantly higher NT-proBNP than men [45–49]. No differences were found between intact and neutered female dogs, as well as between intact and neutered males [44]. In women, NT-proBNP was found to be significantly higher in luteal and follicular phase than in midcycle phase [50]. In humans, the mechanisms behind the influence of gender on natriuretic peptides (NP) are still subject of debate. Initially, the difference between genders was attributed to oestrogens (and, in part, to progesterone), which could have both a direct and indirect stimulatory effect on NPs [47, 50–52]. More recently, the role of gender has been primarily related to testosterone, which has been found to be inversely correlated with NPs likely because of a suppressive effect on their release [45, 50, 53]. However, controversial results have been reported for both oestrogens and testosterone. It is likely that both hormones contribute to the regulation and the determination of NPs levels through the sum of their effects [50]. Even the role of progesterone is not clear because of conflicting results [50, 51, 54–56]. In dogs, no differences were found between intact and neutered female [44], but, to our knowledge, there are no reports about NPs levels in different phase of reproductive cycle and their correlation with sex hormones. Based on the results of the present study, NT-proBNP seems not to be affected by progesterone and not to significantly vary between anoestrus and dioestrus. However, oestrogens and testosterone were not measured, and the other phases of reproductive cycle were not evaluated. Thus, further investigations are warranted in dogs to better define the variation of NPs through the entire cycle and their relationship with sex hormones.

The present study has several limitations. First of all, the small sample size limits the strength and the accuracy of the results; contributors encourage further studies on a large canine population, and even in other breeds, to verify their replicability. Secondly, oestrogens were not evaluated and a possible influence of these hormones on aldosterone could have been missed. However, oestrogens levels in dioestrus are low [17] and their potential effect on RAAS should be limited during this phase. Thirdly, serum sodium and potassium levels can affect RAAS activity [57], and they were not assessed in the present study. Fourthly, assessment of other RAAS components and urinary sodium would help obtain a better understanding of the aldosterone-progesterone relationships. Lastly, although the time of urine collection was restricted between 10 am and 2 pm, an influence of urinary aldosterone and UC circadian variations on the values obtained in the present study cannot be excluded [58, 59].

## Conclusion

These results suggest the existence of a progesterone-aldosterone relationship in canine species, indicating that gender and phase of oestrous cycle should be taken into account for a correct interpretation of aldosterone levels in dogs. Mechanisms underlying this correlation in dogs might be similar to those reported for humans and rodents, that are the MRs antagonism by progesterone with the consequent compensatory RAAS activation, and a direct stimulation of aldosterone production by progesterone as well. Further studies are needed both to confirm these results on a larger canine population and to identify the mechanisms behind this relationship in this species.

## Materials and methods

### Animals and study timeline

This prospective study was conducted in accordance with guidelines of the Animal Welfare Organisation of the Università degli Studi di Milano (approval number 2/ 2016) and with informed consent of the owners. Subjects enrolled in this study were recruited among private owned breeding dogs referred to the Veterinary Teaching Hospital – University of Milan (January-December 2021) for cardiological and reproductive screening/monitoring. All procedures to which patients have been subjected were part of their screening/monitoring program. Adjunctive analyses on serum/plasma were performed on leftover samples, while urine analyses were performed on free-catch samples.

### Inclusion and exclusion criteria

To be enrolled in the study, dogs had to meet the following criteria: Chihuahua breed, intact female and older than 12 months. All dogs had to be either healthy or affected by MMVD ACVIM stage B1^17^. Subjects with any other known disease or subjected to any pharmacological treatment at the time of examinations were excluded.

All dogs included in the study had to undergo two examinations as part of their reproductive monitoring program: the first one during anoestrus (i.e., at basal levels of progesterone) and the second one during following dioestrus (i.e., at high levels of progesterone). During anoestrus, each dog underwent indirect blood pressure measurement, complete physical examination, echocardiography, vaginal cytology, blood sampling (for CBC, biochemistry profile and serum progesterone), collection of a free-catch urine sample (for urinalysis and urinary aldosterone) and assessment of plasma NT-proBNP. In dioestrus, the following procedures were repeated: vaginal cytology, blood sampling (for serum progesterone), collection of a free-catch urine sample (for urinalysis and urinary aldosterone) and assessment of plasma NT-proBNP. Diet information was also collected. Dogs with no abnormalities on medical history and aforementioned procedures were considered healthy. Patients diagnosed with MMVD by echocardiography were classified as stage B1 following the criteria of the ACVIM guidelines [60].

### Systolic arterial pressure and echocardiography

Non-invasive arterial pressure measurements were obtained and interpreted based on published guidelines [61], using a veterinary high-definition oscillatory device 1.

Echocardiography was performed by two experienced echocardiographers, using an ultrasonographic unit (Esaote MyLabOmega) equipped with two multifrequency phased array probes (1–9 and 2–5 Mhz respectively). Each dog underwent a complete echocardiographic examination, in accordance with published standards [62]. All dogs were conscious during the procedure.

### Sample collection, storage and analysis

Blood sampling was performed by venepuncture on fasted dogs (i.e., at least 6–8 h after meal), and blood was immediately collected into EDTA and serum gel tubes.

Blood collected into EDTA tubes was used for CBC. Samples were then immediately centrifugated at 3750 rpm for 5 min and plasma was stored at -80 °C. Refrigerated plasma samples were sent at IDEXX laboratory (Korwestheim, Germany) for plasma NT-proBNP measurement by a second-generation Enzyme-linked Immunosorbent Assay (ELISA)^2^.

Blood collected into serum gel tubes was left to clot at room temperature for 30 min, and then it was centrifugated at 3750 rpm for 5 min. Serum was used for biochemical analysis (urea, creatinine, glucose, total protein, albumin, alanine aminotransferase, alkaline phosphatase). Residual serum was stored at -80 °C and then submitted for the determination of progesterone.

Urine samples were collected by spontaneous micturition and standard urinalysis was performed (i.e., dipstick chemistry test and USG by refractometer). After centrifugation at 1250 rpm for 5 min, supernatant was used for urinary protein and urinary creatinine assessment by Pyrogallol Red Method. Values of UP/UC below 0.5 were considered normal [63]. Residual supernatant was stored at -80 °C for subsequent determination of urinary aldosterone.

Urinary aldosterone concentrations were assessed by a commercially available species-independent ELISA kit 3, following the manufacturer’s recommendations. The ELISA kit was previously validated for the measurement of aldosterone in dog urine after acid hydrolysis [9].

Serum progesterone concentrations were determined by an Enzyme-linked Fluorescent Assay method (Enzyme-linked Fluorescent Assay) 4 validated in dogs [64].

### Statistical analysis

Statistical analysis was performed with commercially available statistical software (IBM SPSS® Statistics 27). The Kolmogorov-Smirnov test was used to assess normality. Parametric variables were reported as mean ± standard deviation (SD) and compared by paired-sample t-test; nonparametric variables were reported as median and interquartile range (IQR) and compared by paired-sample Wilcoxon signed-rank test. To compare a parametric variable with a nonparametric one, paired-sample Wilcoxon signed-rank test was used. Correlations among variables were explored by the Pearson correlation coefficient (ρ), and results were interpreted as follows: ρ ≤ 0.03 weak correlation, ρ > 0.3 and ≤ 0.7 moderate correlation, ρ > 0.7 strong correlation. A P value < 0.05 was considered statistically significant.

A constant value corresponding to half of the limit of detection (LOD) was assigned to patients with serum progesterone and plasma NT-proBNP below the LOD (0.25 ng/ml and 250 pmol/l respectively).

## Data Availability

The datasets used and/or analysed during the current study are available from the corresponding author on reasonable request.

## Footnotes

1 VET-HDO ®- MONITOR, S + B medVET GmbH, Babenhausen, Germany.

2 NT-proBNP: ELISA Cardiopet proBNP test, IDEXX Laboratories, Westbrook, ME.

3 Enzo Life Sciences Aldosterone ELISA kit, Enzo Life Sciences Inc., Farmingdale, NY, USA.

4 MiniVidas automated analyser, bioMérieux, Marcy-l’Étoile, France.

## Abbreviations

RAAS: Renin-angiotensin-aldosterone system
CHF: Congestive heart failure
UAldo: C:Urinary aldosterone-to-creatinine ratio
MR: Mineralocorticoid receptor
BCS: Body Condition Score
CBC: Complete Blood Count
ACVIM: American College of Veterinary Internal Medicine
MMVD: Myxomatous mitral valve disease
NT-proBNP: N-Terminal pro-B-type natriuretic peptide
UP/UC: Urinary protein-to-creatinine ratio
UC: Urinary creatinine
UP: Urinary protein
USG: Urine specific gravity
NP: Natriuretic peptide
SD: Standard deviation
IQR: Interquartile range
LOD: Limit of detection
ELISA: Enzyme-linked immunosorbent assay
